# Profiles of psychopathology and quality of life in individuals with recent displacement experiences: a latent profile analysis

**DOI:** 10.1186/s12889-026-27604-w

**Published:** 2026-04-30

**Authors:** M. Ali Amiri, Gerhard Andersson, Kristofer Vernmark, Anna Leiler, Anna Bjärtå

**Affiliations:** 1 Department of Behavioral Sciences and Learning, Linköping University, Linköping, SE-58183 Sweden; 2 Department of Biomedical and Clinical Sciences, Linköping University, Linköping, Sweden; 3 Department of Clinical Neuroscience, Psychiatry Section, Karolinska Institutet, Stockholm, Sweden; 4 Department of Education, Psychology and Social work, Mid Sweden University, Östersund, Sweden

**Keywords:** Psychopathology, Refugee, Latent profile analysis, PTSD, Quality of life

## Abstract

**Background:**

Mental health conditions such as depression, anxiety, and post-traumatic stress disorder (PTSD) are highly prevalent among forcibly displaced populations. Prevalence rates of common mental health conditions have been studied in refugee groups. However, research is sparse regarding the heterogeneity of psychiatric symptoms and quality of life (QoL) profiles among individuals with recent displacement experiences.

**Methods:**

The current study employed Latent Profile Analysis (LPA) to identify profiles of psychopathology and QoL in a sample of 510 recently arrived refugees in Sweden. The associations of profile membership with socio-demographic factors were thereafter investigated.

**Results:**

Three distinct profiles were identified: a severe psychopathology/low QoL profile (36.27%), a PTSD-dominant/preserved QoL profile (33.14%) and a mild psychopathology/high QoL profile (30.59%). Nationality and residence status were moderately associated with profile membership. Individuals with Afghan nationality were over-represented, and those with Syrian nationality under-represented, in the severe psychopathology/low QoL profile. This association is likely explained by residence status: 82% of individuals in the severe psychopathology/low QoL profile lacked a residence permit, with only 3.6% of Afghans having received a residence permit, compared to 59.1% of Syrians.

**Conclusions:**

The results underscore the heterogeneity of psychopathological symptoms and QoL in individuals with recent displacement experiences, as well as a significant influence of contextual factors like residence status on their mental health and QoL. These findings may have implications for informing psychological treatments and migration policies.

**Supplementary Information:**

The online version contains supplementary material available at 10.1186/s12889-026-27604-w.

## Introduction

The experience of forced displacement and becoming a refugee is often accompanied by numerous physical and mental health risks [1, 2]. Throughout the journey to a new settlement country, refugees endure significant losses and can be exposed to traumatic events, such as physical and sexual violence, kidnapping for ransom, arbitrary detention, robbery, and human trafficking [2]. Many have also faced challenges beginning before the journey, as refugees often experience war and direct exposure to violence, persecution, mass killings, and torture in their country of origin [3, 4]. Furthermore, once they reach their final destination, feelings of loneliness and uprootedness, coupled with experiences of social exclusion, discrimination, precarious legal status, and unemployment, further undermine their health and well-being [5–7].

Despite belonging to a vulnerable and burdened group, refugees are often able to cope with the hardships [8, 9]. They can develop resources such as strength and adaptability to maintain their psychological stability after adverse and traumatic experiences and demonstrate a remarkable resilience. Resilience is defined as “the capacity of a dynamic system to successfully adapt to disturbances that threaten the viability, function or the development of that system” [10]. Resilience at personal [11] and family levels [12] has consistently been identified as a protective factor against mental health problems. Nevertheless, the cumulative impact of pre-, peri-, and post-migration factors significantly challenge refugees’ capacity for resilience and are strongly associated with mental health conditions such as post-traumatic stress disorder (PTSD), depression, and anxiety [6, 13–15]. Research suggests that higher exposure to pre-migration traumatic experiences and after migration stress are the most consistent factors associated with PTSD, depression and anxiety [16]. Therefore, prevalence rates of these mental health disorders among refugees resettled in high-income countries are substantially higher compared to non-refugee populations worldwide [17]. In addition to common mental health disorders, physical pain [18] and physical health problems are also frequently reported by refugees [19, 20]. The prevalence of physical health problems is nearly doubled in refugees with comorbid PTSD and depression, compared to refugees without either conditions [21], highlighting the impact of mental health disorders on physical health.

Mental health problems have also been identified as a key factor driving lower health-related quality of life in refugee samples [22]. Studies with refugees indicate that suffering from general psychological distress and mental disorders (i.e., depression, anxiety and PTSD) strongly reduces quality of life (QoL) [23–25]. To this end, research suggests that the level of QoL in refugee populations is below international population norms [26] and below the general population of the hosting countries [22, 27]. The World Health Organization (WHO) defines QoL as “individuals’ perceptions of their position in life in the context of the culture and value systems in which they live and in relation to their goals, expectations, standards and concerns” [28]. Given that culture and contextual factors significantly influence the expression of mental health symptoms [29], individuals’ perceptions of themselves within their culture and living context may shape both their psychopathological symptoms and their coping strategies. As QoL measures encompass dimensions that go beyond what symptom-based measures capture, they can be a meaningful complement in psychological research and practice [30]. Therefore, assessing QoL alongside psychopathological symptoms aligns with a holistic approach to health as “not merely the absence of disease, but physical, mental, and social well-being” [31].

Many different socio-demographic factors associated with poor mental health and decreased QoL among refugees have been identified, including unemployment [32], uncertain asylum status [33–36], living in camps or shared asylum accommodation [27, 34, 36] and family separation [34, 36, 37]. Research also suggests that female gender [23, 38–40], and low and medium educational level compared with higher educational level [40] increase the risk of common mental health disorders (CMHD) among refugees. Moreover, older age is associated with higher risk of PTSD [41], and higher levels of comorbid depression, anxiety and PTSD symptoms [42].

On the other hand, having an established network, successful social integration [25, 43], male gender, younger age [43] and employment [32, 34] are suggested factors associated with better health and QoL in refugees. Research suggests a difference in QoL depending on the amount of time passed since arriving in a new country. For example, recently resettled asylum seekers (less than 6 months) reported higher rating of their QoL than those who lived in the country for at least two years [44], highlighting the adverse effect of long asylum procedure on QoL. These findings motivate further investigation of daily post-migration stressors.

Psychopathology has predominantly been studied through variable-centered approaches, assuming that the sample is drawn from a single population with a shared set of averaged parameters [45]. Thus, analytical approaches usually entail grouping similar variables together, examining the effect of one variable on another, or analyzing the association between variables [46, 47]. Given the individual differences in the presentation of mental health disorders, common variable-oriented methods do not allow for inference about the single person and individual patterns of functioning [48]. On the other hand, person-centered approaches framed within the holistic-interactionist research paradigm, see [48, 49], view the individual as an integrated whole, where various elements work together dynamically to create a functional system. This approach emphasizes analyzing patterns of information, rather than separate variables, and seeks to identify typical patterns shared by subgroups within a total sample [48]. When studying psychopathology, the application of a person-centered approach focuses on identifying typical patterns of symptoms and behaviors across individuals, rather than assuming uniformity in mental health presentations [50].

Mental health outcomes differ markedly among refugees [51]. Although refugees may experience similar types of trauma, their mental health outcomes and QoL are influenced by unique combinations of personal, social, and contextual factors [52, 53]. Given the complex interplay of pre-, peri-, and post-migratory factors, a person-centered analytical approach may help identify subgroups that differ significantly in their psychopathological and well-being profiles [54].

Previous LPA studies on refugees’ mental health have included measures of psychopathology as class indicators and treated QoL as an outcome, for example [55]. Including QoL as an indicator within the same LPA model will alter the latent structure of the model, this means that profile enumeration will be driven jointly by variation in symptoms and QoL. This will increase the likelihood of identifying subgroups differing not only in psychopathology but also QoL that would not emerge in a symptom-only specification. Moreover, although QoL is often associated with psychopathological symptoms under linear assumptions [24, 26], research also indicates that these constructs frequently exhibit inconsistent patterns of change. For instance, in a meta-analysis reductions in depression symptoms did not necessarily correspond to improvements in QoL, and vice versa [56]. Therefore, an LPA model that includes both psychopathology and QoL will enable to find discordant profiles that help explain discrepancies in patterns of change, patterns that may not be explained through linear assumptions. When studying culturally diverse populations such as newly arrived refugees, LPA can be used to identify distinct latent profiles based on mental health symptoms and QoL, offering a more holistic understanding of their mental health and overall well-being. Identifying such profiles also informs targeted interventions tailored to the needs of different subgroups, supporting more effective resource allocation and treatment designs [52].

The aim of the current study was to identify subgroups of individuals with recent displacement experiences with respect to psychopathology and QoL, and to examine how profiles differ in relation to socio-demographic variables including gender, age, nationality, education, employment status, accompaniment, residence permit status, and housing situation.

## Method

### Participants and procedure

The study employed a cross-sectional, exploratory design using convenience sampling. The target population consisted of all adult refugees (18 years and older) living in the region Jämtland-Härjedalen in Sweden in the period of November 2016 to April 2017. The sample included both asylum seekers and individuals who had recently been granted asylum but were awaiting relocation to a municipality (lists were provided by the migration agency). The project was approved by the Regional Ethics Board of Jämtland-Härjedalen (2016-364-31). Given that refugees are a hard-to-reach group [57], participants were recruited through refugee housing facilities in the region, with the assistance of facility managers and volunteers in close contact with refugees. Postal invitations translated into the five most common languages spoken among refugees in the region (Arabic, Dari, Farsi, Somali, and Tigrinya) were sent to all adult refugees, inviting them to complete the questionnaires online or attend in-person screening meetings. A follow-up letter was sent to all refugees in the region, regardless of mother tongue, and to those who had missed the initial screening, inviting them to participate in the study.

The total sample included 1332 individuals, but 577 (43%) participated in the study. After excluding 67 individuals with incomplete responses, 510 (38% of the sample) were included in the final analysis (see Table 1 for demographic characteristics). Data were collected through self-administered questionnaires using Qualtrics software (Qualtrics; Provo, UT, 2005). Participants who attended the screening sessions were provided with I-Pads to complete the survey. Audio support was offered in Arabic, Dari, Farsi, and Tigrinya. Bilingual staff members, were present to provide clarification and assistance as needed. All materials were translated into the five common refugee languages in the region as well as into English and Swedish. More details on the translation procedures are described in [26, 58]. Previous studies on this data have not employed LPA.

**Table 1:** Sociodemographic characteristics of the participants

Characteristics	*N* = 510	
	*n*	%
Gender		
Male	367	72.0
Female	136	26.6
Other	7	1.4
Nationality		
Afghanistan	196	38.4
Syria	137	26.9
Iraq	51	10.0
Iran	22	4.3
Eritrea	21	4.1
Somalia	11	2.2
Other^a^	72	14.1
Age groups		
18–25	163	32.0
26–35	200	39.2
36–45	87	17.0
46–55	45	8.8
56–65	12	2.4
66+	3	0.6
Highest educational level		
No high school education	298	59.1
High school diploma	73	14.5
Vocational diploma	2	0.4
University	9	1.8
Higher education diploma	57	11.3
Bachelor’s degree	35	6.9
Master’s degree	29	5.8
PhD	1	0.2
Residence permit		
Yes	143	28.0
No	367	72.0
Employment status		
Student	92	18.4
Employed	289	57.9
None	118	23.6
Accompaniment		
Accompanied	291	57.3
Unaccompanied	217	42.7
Living in asylum accommodation		
Yes	454	89.1
No	56	10.9

^a^Other (*N* < 5) = Palestine, Ethiopia, Pakistan, Algeria, Morocco, Nigeria, Egypt, Kuwait, Lebanon, Sudan, and Yemen

### Indicators

#### Patient health questionnaire-9

The patient health questionnaire 9-item scale (PHQ-9) is a self-administered instrument assessing the severity of depressive symptom in primary care [59]. The questionnaire consists of nine items rated on a four-point Likert scale (0–3), with total scores ranging from 0 to 27. Respondents are asked to rate how frequent they have been bothered by a given symptom from “*Not at all*” (scored as 0) to “*Nearly every day*” (scored as 3) over a course of two weeks. Normative increasing severity range for this instrument was set to 0–4 for minimal depression, 5–9 for mild depression, 10–14 for moderate, 15–19 for moderately severe, and 20–27 for severe depression. The internal consistency of PHQ-9 has been reported as excellent with a Cronbach’s α of 0.89 and test-retest reliability of 0.84 [59]. In the current sample the internal consistency was Cronbach’s α = 0.87.

#### Generalized anxiety disorder scale-7

The generalized anxiety disorder 7-itm scale (GAD-7) is a self-administered instrument used to screen for generalized anxiety disorder [60] and overall anxiety [61]. Respondents are asked to report how often they have been bothered by an anxiety pertaining symptom from “*not at all* = 0”, “*several days* = 1”, “*more than half the days* = 2” and *nearly every day* = 3” over a course of two weeks. Total scores range from 0 to 21 with a severity level of 0–4 indicating (minimal), 5–9 (mild), 10–14 (moderate), and 15–21 (severe) anxiety [60]. The internal consistency of GAD-7 has been reported as excellent with a Cronbach’s α = 0.92. The test-retest reliability has been reported as good with an intra class correlation = 0.83. In the current sample the internal consistency was Cronbach’s α = 0.91.

#### Primary care post traumatic stress disorder scale

The primary care PTSD 4-item (PC-PTSD) is a screener for PTSD in primary care settings [62]. This self-administered scale has four items assessing four characteristic symptoms (intrusion, avoidance, hyper-arousal, and emotional numbing) related to a traumatic event. Participants respond with “yes” or “no,” and the total number of “yes” responses is summed. Although, a total score of 3 is recommended as the cut-off for detecting PTSD, but it is also recommended to further assess patients with a score of 2 in primary care settings [62]. Despite its brevity, PC-PTSD has demonstrated high efficiency in identifying PTSD diagnoses, comparable to longer scales like the PCL [63]. In the current sample the internal consistency was Cronbach’s α = 0.77.

#### World health organization quality of life-brief version

The World Health Organization Quality of Life-Brief Version (WHOQOL-BREF) is a transcultural measure of QoL and health. It is a self-reported instrument consisting of 24 items plus 2 additional items that measure individual perceptions of global QoL and health status, using a five-point Likert scale ranging from 1 to 5 [64]. The WHOQOL-BREF is a brief version of the WHOQOL-100 [28] and assesses four domains: physical health, psychological health, social relationships, and environmental factors. The mean score in each domain reflects an individual’s perception of their satisfaction with that aspect of life, with higher scores indicating better perceived QoL. The internal consistency of the WHOQOL-BREF in a population of 11,830 was reported as acceptable, with Cronbach’s α = 0.77 [65]. In the current sample, internal consistency was Cronbach’s α = 0.92.

We aimed to use QoL as a general measure of individuals’ perceived functioning and well-being in their post-migratory context. Therefore, the sum of transformed (4–20) scores from all four domains of the WHOQOL-BREF was used in the analysis due to high intercorrelation between the domains, for correlations see (Table S1 in the Supplementary Material). In LPA, it is also suggested to minimize the number of multicollinear variables as they are likely to contribute to poorly converging models [66]. While it seemed feasible to use only item 1 on WHOQOL-BREF which assesses self-perceived QoL, the developers recommend that all four domains should be considered when evaluating overall QoL [64]. Moreover, the correlations between WHOQOL-BREF domain scores and the total score ranged from *r* = .80-0.85 indicating strong correlation. Sum of the all domain scores has been used in previous studies as well, for details see [67, 68].

### Statistical analysis

A latent profile analysis was conducted with the inclusion of PHQ-9, GAD-7, PC-PTSD and WHOQOL-BREF as main indicators. We used the total scores of all these continuous measures to better approximate them in the models [47]. First, included indicators were assessed for normality, outliers, and missing values. Descriptive statistics (*M* and *SD*) and bivariate correlations (Pearson’s *r*) of the main study variables were calculated. Latent profile models were estimated using standardized scores of the main indicators. Finally, posterior probabilities associated with latent profiles were extracted and most likely profile membership was used in chi-square tests of association to analyze the association between socio-demographic variables and latent profiles’ membership. Chi-square tests were conducted using complete cases, with missing data handled via list-wise deletion. The adjusted standardized residual (ASR) method was used to identify specific cells making significant contribution to the chi-square test results [69]. Residuals greater than |2.0| indicated significant discrepancies between observed and expected frequencies in the study’s contingency table.

#### Latent profile analysis (LPA)

Latent profile analysis (LPA) and latent class analysis (LCA) are probabilistic modelling algorithm to classify groups of individuals according to some constructs that are not directly measurable [70, 71]. Both LPA and LCA are mixture models for cross-sectional data with the main difference of LPA being applied to continuous response variables whereas LCA applies to categorical ones [47, 70]. In finite mixture modeling like LPA/LCA the unobserved or latent groups are inferred from patterns of the individual set of observed responses to the indicators [66, 71], which allows to group similar people together unlike factor analysis which groups items [71]. Moreover, in LPA, as a “person-centered approach”, individuals are assigned to profiles based on probabilities [72, 73], making it conceptually advantageous over the “deterministic” assignments used in traditional cluster analysis [47]. A detailed account of latent variable mixture models is found in [72].

LPA was performed in the R statistical environment [74], employing the “mclust” [75] and “tidyLPA” [76] packages. The mclust package in R allows for flexibility in modelling quantitative data with several covariance structures and different numbers of mixture components [72]. Concerning parameter restrictions to increase model parsimony and estimation ability; mclust was allowed to fit a variety of models with different parameter restrictions as this data-driven strategy accounts for the priori suggested restrictions of parameters [47]. Then, the best fitting model, including the number of latent profiles was selected. For more detailed information on the multivariate models, including how model parameters can freely vary or be constrained across profiles, readers are referred to [47, 77]. Subsequent to selecting the best fitting model for the indicators, the tidyLPA package was used to obtain additional fit indices for a better comparison between competing models in a reiteration process starting with 1 to 6 profiles retaining the suggested model configuration.

To select the optimal model the Bayesian Information Criteria (BIC), Sample Adjusted BIC (SABIC) and Akaike information criteria (AIC) were obtained as the most commonly used fit indices e.g. [73, 78]. Considering the absolute values for the fit indices explained by [79], low values on all information criteria (BIC, AIC, and SABIC) indicate better model fit [47]. In addition to information criteria, models with adjacent number of latent profiles were compared by the Bootstrapped Likelihood Ratio Test (BLRT) as an effective measure in class enumeration [80]. Entropy was used for classification accuracy, measuring the certainty of class separation [66]. It ranges from 0 to 1, with higher values indicating a model’s strong ability to distinguish between model components and their distinctiveness. In addition to fit indices, model interpretability and conformance to theory [72], the relative size of the emergent profiles was informed by the criterion that each profile should comprise more than 5–8% of the sample [71] .

For details on the categorization of socio-demographic variables, see the Supplementary Material (Categorization of Socio-Demographic Variables section).

## Results

Data were normally distributed across selected indicators, and no extreme outliers were detected. Three missing completely at random (MCAR) cases in the WHOQOL-BREF were handled utilizing the “Mice” package in R [81], using the “predictive mean matching” method. Pearson’s bivariate correlation showed moderate to very strong relationships among all mental health (i.e., depression, anxiety and PTSD) and QoL indicators (all *p*s < 0.01). QoL was negatively correlated with psychopathological measures (see Table 2).

**Table 2:** Means, standard deviations, and correlation coefficients for the indicators used in the latent profile analysis

Variable	M	SD	1	2	3	4
1. GAD_7	9.43	6.20	-	0.79^**^	0.57^**^	− 0.47^**^
2. PHQ_9	11.53	6.87		-	0.50^**^	− 0.51^**^
3. PC_PTSD	2.46	1.38			-	− 0.32^**^
4. WHOQOL_BREF	49.00	10.82				-

*Abbreviations*: *PHQ-9* Patient Health Questionnaire 9-items scale, *GAD-7* Generalized Anxiety Disorder 7-item scale, *PC-PTSD* Primary Care PTSD, *WHOQOL-BREF* World Health Organization Quality of Life—brief version

**p < .01

### Latent profiles

Among the inspected models with various parameter restrictions, the best fitting solution was a three-profile model with varying means and equal variances and covariances across profiles. For the BICs of different variance parametrization (see Figure S1 in the Supplementary Material). Table 3 presents model estimates and profile comparisons for the one to six profile solutions. Although, the five and six profiles showed lower SABIC and AIC values, the smallest class comprised less than 5% of the sample in the five-profile solution and just above 5% in the six-profile solution, and both models showed lower entropy. In addition, visual inspection indicated overlapping profiles with high within class variabilities (Figure S2 in the Supplementary Material).

The three-profile solution showed a lower BIC and higher entropy relative to competing models. The BLRT was also significant for the three-profile model (*p* < .05), and the non-significant BLRT for the four-profile indicated that adding an additional profile did not improve model fit. Average posterior probabilities for the three-profile solution were 0.92, 0.82, and 0.90 for profile1-3, respectively. This was above the recommended threshold level of 0.80 [73] for each profile. Overall, the three-profile solution demonstrated a better parsimony, clear profile separation, and interpretability, therefore this was the final selected model. A random split-sample validation (70%/30%) was conducted to examine the stability of the solution. The three-profile model was supported in both subsamples based on BIC, entropy and BLRT (see Table S3 in the Supplementary Material). Profile shapes and class proportions were broadly comparable across subsamples.

**Table 3:** Fit indices for the six tested profiles

Number of profiles	AIC	BIC	SABIC	Entrp	Prob min	Prob max	*N* min	*N* max	BLRT_ val	BLRT_ *p*
1 profile	11510.60	11569.88	11525.44	1.00	1.00	1.00	1.00	1.00	*NA*	*NA*
2 profiles	11455.60	11536.05	11475.74	0.72	0.72	0.93	0.40	0.59	65.00	0.01
**3 profiles**	**11363.03**	**11464.66**	**11388.48**	**0.74**	**0.74**	**0.92**	**0.30**	**0.36**	**102.56**	0.01
4 profiles	11371.00	11493.80	11401.75	0.65	0.65	0.88	0.09	0.37	2.03	0.60
5 profiles	11349.46	11493.43	11385.51	0.70	0.70	0.87	0.02	0.32	31.54	0.01
6 profiles	11342.07	11507.22	11383.42	0.72	0.72	0.88	0.06	0.22	17.39	0.03

*Abbreviations*: *AIC* Akaike information criterion, *BIC* Bayesian information criterion, *SABIC* sample size-adjusted Bayesian information criterion, *Entrp*, Entropy, *Prob min/max*, the average latent class probability for most likely class membership by assigned class, *N min*/*max* proportion of the sample assigned to the smallest or largest classes, *BLRT_val*, Bootstrapped likelihood ratio test, *BLRT_p* P-value for the bootstrapped likelihood ratio test. Selected LPA model is boldfaced in the table

**Fig. 1:**
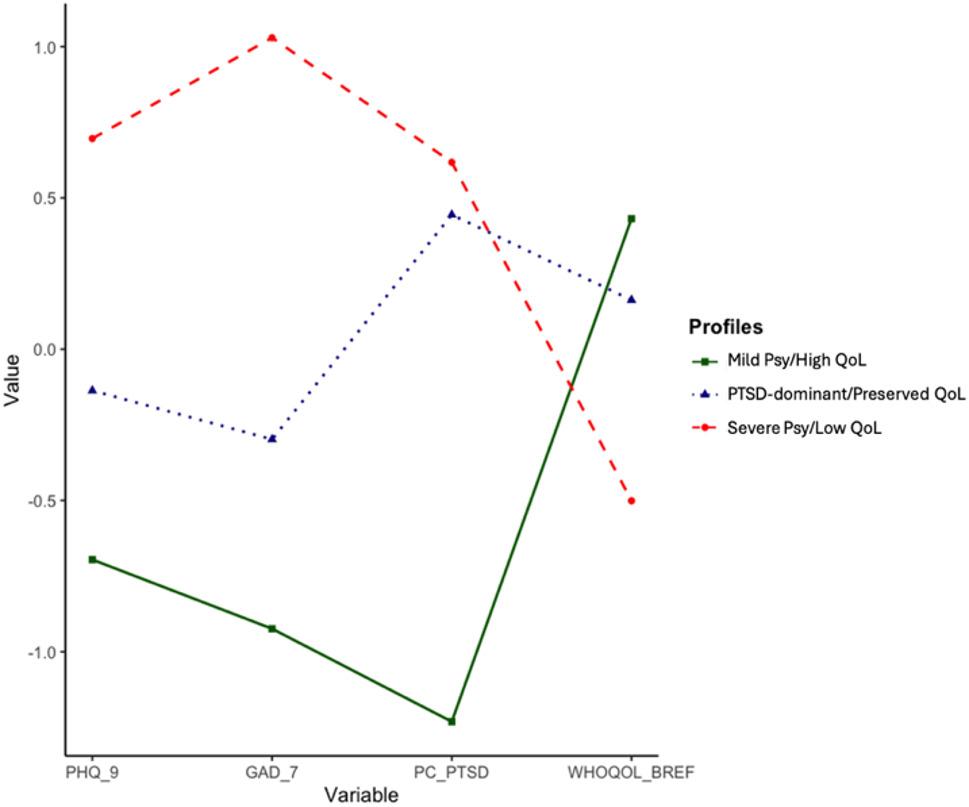
Latent profiles of psychopathological symptoms and Qol in individuals with recent displacement experiences. Abbreviations: PHQ-9 Patient health questionnaire 9-item scale, GAD-7 Generalized anxiety disorder 7-item scale, PC-PTSD Primary care PTSD, WHOQOL-BREF World health organization quality of Life—brief version. Values are standardized

Figure 1 presents the standardized mean scores relative to the grand mean in the sample for the participants in each of the three profiles, showing that the profiles differ in the degree of psychopathological symptoms severity and QoL. The first profile encompassed 156 participants (30.59%). This profile exhibited below average scores in all psychopathological indicators (depression, anxiety and PTSD) and above average scores in the QoL indicator. We refer to this profile as *Mild psychopathology/High Quality of Life (Mild Psy/High QoL)*. The second profile accommodated 169 participants (33.14%). This profile scored below average in depression and anxiety, and above average in PTSD and QoL. This profile was named *PTSD dominant- Preserved Quality of Life (PTSD-dominant/Preserved QoL)*. The third profile consisted of 185 participants (36.27%) who scored above average in all psychopathological indicators and below average in QoL. This profile was named *Severe Psychopathology/Low Quality of Life (Severe Psy/Low QoL)*.

Pairwise comparisons with Tukey correction between three latent profiles indicated that all three profiles were significantly different from each other in depression, anxiety and QoL at *p* < .001 level. For PTSD symptoms, the difference between PTSD-dominant/Preserved QoL and Severe Psy/Low QoL profiles was significant at *p* < .05 (see Table S2 in the Supplementary Material).

Estimated means and standard deviations for the indicators in each profile is presented in Table 4. The majority of participants in the Severe Psy/Low QoL profile endorsed a clinical level of symptom severity for PTSD, anxiety and depression based on the conventional cut-off scores of the respective outcome measure. Individuals in the Severe Psy/Low QoL profile also showed substantially low QoL.

**Table 4:** Means and standard deviations of the indicators for the three latent profiles

	Profile 1: Mild Psychopathology/High QoL (*n* = 156)		Profile 2: PTSD-dominant/Preserved QoL (*n* = 169)		Profile 3: Severe Psychopathology/Low QoL (*n* = 185)	
	M	SD	M	SD	M	SD
PHQ-9	6.72	5.45	10.36	5.48	16.68	5.55
GAD-7	3.50	3.43	7.49	2.97	16.21	3.00
PC-PTSD	0.74	0.76	3.10	0.75	3.34	0.83
WHOQOL-BREF	53.98	10.06	50.72	9.28	43.21	10.14

*Abbreviations*: *PHQ-9* Patient Health Questionnaire 9-item scale, *GAD-7* Generalized Anxiety Disorder 7-item scale, *PC-PTSD* Primary Care PTSD, *WHOQOL-BREF* World Health Organization Quality of Life—brief version

### Associations between latent psychopathological and QoL profiles and socio-demographic characteristics

Table 5 presents percentages of the eight socio-demographic indicators in each latent profile. The Chi-square test for association indicated a moderate association between profile membership and residence permit, χ^2^(2, *N* = 510) = 39.56, *p* < .001, [Cramer’s *V* = 0.28]. Accordingly, more individuals without a residence permit were observed in the Severe Psy/Low QoL and PTSD-dominant/Preserved QoL profiles (41% and 36% respectively). Conversely, the number of individuals who had obtained a residence permit were higher in the Mild Psy/High QoL profile (51%) compared to the PTSD-dominant/Preserved QoL and Severe Psy/Low QoL profiles (25% and 24% respectively). The proportion of individuals without a residence permit was significantly larger within the Severe Psy/Low QoL 82% (95% CI [0.75, 0.87]) and PTSD-dominant/Preserved QoL profiles 79% (95% CI [0.72, 0.85]) as compared to the Mild Psy/High QoL profile 53% (95% CI [0.45, 0.61]).

**Table 5:** Number and percentage of socio-demographic variables in each latent profile

Indicators	Mild Psy/High QoL profile	PTSD- dominant/Preserved QoL profile	Severe Psy/Low QoL profile
Age			
18–25	39 (24%)	60 (37%)	64 (39%)
26–35	65 (33%)	72 (36%)	63 (32%)
36–65	52 (36%)	36 (25%)	56 (39%)
Gender			
Female	43 (32%)	34 (25%)	59 (43%)
Male	113 (31%)	132 (36%)	122 (33%)
Employment status			
Unemployed	28 (24%)	36 (31%)	54 (46%)
Employed	124 (33%)	129 (34%)	128 (34%)
Residence permit			
No RP	83 (23%)	133 (36%)	151 (41%)
RP	73 (51%)	36 (25%)	34 (24%)
Accompaniment			
Unaccompanied	66 (30%)	74 (34%)	77 (35%)
Accompanied	89 (31%)	94 (32%)	108 (37%)
Accommodation			
Asylum accommodation	135 (30%)	153 (34%)	166 (37%)
Else	21 (38%)	16 (29%)	19 (34%)
Education			
With higher education	49 (37%)	43 (33%)	39 (30%)
Without higher education	103 (28%)	124 (33%)	144 (39%)
Nationality			
Afghanistan	28 (14.3%)	72 (36.7%)	96 (49%)
Syria	61 (44.5%)	48 (35%)	28 (20.4%)
Iraq	14 (27.5%)	19 (37.3%)	18 (35.3%)
Iran	6 (27.3%)	5 (22.7%)	11 (50%)
Eritrea	14 (66.7%)	3 (14.3%)	4 (19%)
Somalia	3 (27.3%)	6 (54.5%)	2 (18.2%)
Other ^a^	30 (41.7%)	16 (22.2%)	26 (36.1%)

*Note. RP* Participants with a residence permit, *No RP* Participants without a residence permit, *Else* participants not living in asylum accommodation

^a^Other nationality groups (N < 5)

Nationality yielded a moderate association with profiles’ membership χ^*2*^(12, *N* = 510) = 68.15, *p* < .001, [Cramer’s *V* = 0.25]. Individuals with Afghan nationality were over-represented in the Severe Psy/Low QoL profile (49%) and under-represented in the Mild Psy/High QoL profile (14.3%). Conversely, refugees with Eritrean and Syrian nationalities had higher representation in the Mild Psy/High QoL (respectively 66.7% and 44.5%). Individuals with Syrian nationality had lower representation in the Severe Psy/Low QoL profile (20.4%) for details see (Table S4 in the Supplementary Material).

The employment status variable showed a weak association with the latent profiles’ membership, χ^2^(2, *N* = 499) = 6.26, *p* < .05, [Cramer’s *V* = 0.11], with more unemployed participants in the Severe Psy/Low QoL profile (46%), compared to both other profiles (respectively, 31% and 24%, for PTSD-dominant/Preserved QoL and Mild Psy/High QoL profiles). The association of gender with profiles’ membership was also weakly significant, χ^2^(2, *N* = 503) = 6.47, *p* < .05, [Cramer’s *V* = 0.11], with more females in the Severe Psy/Low QoL profile (43%), whereas more males were observed in the PTSD-dominant/Preserved QoL profile (36%).

The age groups showed a weak association with the three latent profiles, χ^2^(4, *N* = 507) = 9.98, *p* < .05, [Cramer’s *V* = 0.10]. Accordingly, fewer young participants (ages 18–25) were associated with the Mild Psy/High QoL profile (24%), and fewer old adults (ages 36–65) were associated with the PTSD-dominant/Preserved QoL profile (25%). Standardized adjusted residuals are presented in (Table S4 in the Supplementary Material).

To investigate dependencies between associated factors, we conducted a contingency analysis between two variables (nationality and residence permit) that showed moderate associations to profiles’ membership. The association between nationality and residence permit was significant *χ*^*2*^(6, *N* = 510) = 183.24, *p* < .001, [Cramer’s *V* = 0.6]. Thus, more individuals with Afghan, Iraqi, and Iranian nationalities (96.4%, 96.1% and 95.5%, respectively) had not received a residence permit whereas more people with Syrian and Eritrean nationalities (59.1% and 71.4%, respectively) had received a residence permit (see Table S5 in the Supplementary Material for details).

## Discussion

The latent profile analysis (LPA) identified three distinct profiles. A profile with severe psychopathological symptoms and low QoL (36.27%), a PTSD-dominant psychopathological symptoms with preserved QoL profile (33.14%), and a profile characterized by low psychopathology (i.e., depression, anxiety and PTSD symptoms) and high QoL (30.59%) emerged. All three profiles significantly differed from each other based on the severity of depressive, anxiety, and PTSD symptoms as well as the levels of QoL. From the socio-demographic factors, residence status and nationality showed greater associations with profile membership, denoting individuals without a residence permit were over-represented in the Severe Psy/Low QoL and the PTSD-dominant/Preserved QoL profiles. Whereas individuals with a residence permit were over-represented in the Mild Psy/High QoL profile. Concerning nationality, individuals with Afghan nationality were over-represented in the Severe Psy/Low QoL profile and under-represented in the Mild Psy/High QoL. Inversely, more individuals with Syrian nationality were represented in the Mild Psy/High QoL profile and less in the Severe Psy/Low QoL profile.

The three latent profiles of psychopathology identified in the current study extends on previous studies identifying three subgroups of psychopathology among treatment-seeking refugee [82] and non-refugee populations [83, 84]. The emergence of the Severe Psy/Low QoL- the largest profile and the Mild Psy/High QoL- the smallest profile in the current study is aligned with previous LPA/LCA studies on comorbid mental health symptoms in refugee populations. For example [82], identified three latent profiles of moderate (10.2%), severe (43.0%) and highly severe (45.9%) PTSD, depression, anxiety and somatic symptoms in a sample of 1147 treatment-seeking refugees. In a nationally representative sample of refugees resettled in Australia [42], identified five subclasses (pervasive symptom − 19.2%, high PTSD symptom − 17.1%, high depression/anxiety symptom − 16.4%, moderate PTSD symptom − 16.2%, and low symptom − 31.1%). Our findings accord with those of [42] by identifying a mild and a severe depressive, anxiety and PTSD symptom profiles. In the current study, participants in the Severe Psy/Low QoL profile- the largest profile, endorsed the highest symptoms level in depression, anxiety and PTSD. This is consistent with previous non-LPA studies suggesting high prevalence of mental health problems among refugees [17, 26, 41].

We also identified a distinct subgroup of participants with below average depressive and anxiety symptoms, above average QoL and high PTSD symptoms, (the PTSD-dominant/Preserved QoL profile). This suggests that there is a subgroup of refugees who despite suffering from high PTSD symptoms, demonstrate resilience and maintain their QoL. Therefore, depressive and anxiety symptoms are moderate among them. Previous research suggests that higher PTSD is associated with poor QoL [23–25, 85]. Nevertheless, high QoL (above average) despite high PTSD symptoms in this subgroup can be an indication of heterogeneity among refugee groups in terms of symptom manifestation and coping. Our finding is consistent with research suggesting various responses to a traumatic event [86, 87] as well with those advocating the co-existence of resilience with psychopathology [88]. This research trend argues that even in severe PTSD cases some of the most resilient people do not succumb to the negative effects of their condition [89]. In the current study, as we did not assess traumatic experiences, we refrained from interpreting mild levels of anxiety, depression, and PTSD symptoms observed in the Mild Psy/High QoL profile as indicators of resilience. First, because we defined resilience as “the ability to bounce back from stressful events”, and previous research indicates that resistance to adversity is not the same as developing symptoms and bouncing back [88, 89]. Second, most of the individuals in this profile had obtained a residence permit; a factor associated with reduced psychopathological symptoms and increased health-related QoL [90]. Instead, we interpreted high QoL in the presence of elevated PTSD and moderate anxiety and depressive symptoms as an indication of coping with trauma and ongoing stressors, such as insecure residence status. However, in other LCA-studies for example, in [91, 92] low probabilities of PTSD and prolonged grief disorder (PGD) symptoms were considered resilience.

Most of the previous LPA-studies with refugees have focused on potentially traumatic events (PTEs) and associated psychopathological symptoms. Some have studied comorbid mental health conditions such as depression and anxiety [42, 55], prolonged grief disorder [91, 92], PTSD symptoms [93] and somatic symptoms [55] and have reported findings with subgroups varying. All these studies were conducted with community samples or groups of refugees with longer settlement history. Given the influences of post-migration stressors on the mental health of individuals with recent migration experiences [5, 82, 93], the sample in the present study shows important differences in characteristics compared to settled refugees in a host country.

Research suggests that mental health problems in forcibly displaced individuals are not only predicted by life-threatening events but also from ongoing threats or post-migration stress [4, 5, 42, 53]. In our study, the Severe Psy/Low QoL profile encompassed mostly participants without a residence permit (*n* = 151, 81.6% of members), predominantly refugees with Afghan nationality (*n* = 96, 51.9% of members). Important to highlight is that only a few refugees with Afghan nationality (*n* = 7/189, 3.6%) reported that they had received a residence permit (see Table S5 in the Supplementary Material for details). Previous research evidences that insecure residence status is associated with high rates of psychopathology including depression, anxiety and PTSD [33, 35, 94, 95] and lower QoL, e.g. [26]. In a study, those with insecure visa status were five times more likely to experience high levels of depressive and anxiety symptoms [94]. This finding suggests that while there might be discrepancies in terms of severity and types of psychopathological symptoms and levels of QoL among refugee groups, uncertain residence status seems to have a significant deleterious impact on refugees’ well-being and QoL.

The Mild Psy/High QoL profile accommodated the largest percentage of individuals with residence permit (*n* = 73, 47% of profile members), suggesting that lower psychopathological symptoms (i.e., depression, anxiety and PTSD) and high QoL may be associated with the security of having received a residence permit. In the present study refugee groups with a higher proportion of members who had received a residence permit, such as Syrian (*n* = 81, 59.1%) and Eritrean (*n* = 15, 71.4%) nationalities were over-represented in the Mild Psy/High QoL. On the significant role of residence status on psychopathological symptoms, also [90] have found a reduction in depression and PTSD symptoms and an increase in health-related QoL after a visa conversion status (from temporary visa to permanent residency) in Mandaean refugees. Therefore, high PTSD symptoms in the PTSD-dominant/Preserved QoL profile in the current study could possibly be due to uncertain residence situation, as 78.7% (*n* = 133) of individuals associated with this profile had not obtained residence permit at the time of this study.

Drawing upon previous research, we found weak associations between employment status, gender, age and the three obtained latent profiles. Consistent with [32] suggesting that unemployment is associated with poor mental health among refugees, in our study more unemployed individuals were represented in the Severe Psy/Low QoL profile. In previous studies e.g. [23, 39, 40], female gender is suggested as a risk factor for common mental health disorders among refugees. Similarly, in our study more females were associated with the Severe Psy/Low QoL profile whereas more males were represented in the PTSD-dominant/Preserved QoL profile. Regarding age, our results showed fewer young adults (ages 18–25) in the Mild Psy/High QoL profile while they were not significantly represented in PTSD-dominant/Preserved QoL or Severe Psy/Low QoL profiles. In relation to this finding, evidence suggests higher incidences of depression and PTSD in refugee minors ([96]). Additionally, less old adults (ages 36–65) were associated with PTSD-dominant/Preserved QoL profile but not over- or under-represented in other profiles. Given that PTSD-dominant/Preserved QoL profile is characterized by high PTSD symptoms and moderate depression and anxiety, this finding is contradicting some previous findings suggesting that old age is associated with higher risk of PTSD [41] and PTSD, depression and anxiety together [42]. Our finding on this variable could have potentially been influenced by the nature of our data collection. We collected age in a categorical manner containing a broad age interval from 36 to 65 years. This finding needs to be investigated through longitudinal data and sensitive analyses.

### Strengths and Limitations

To our knowledge, this is the first LPA-study investigating subgroups of newly arrived refugees driven from a heterogeneous sample based on psychopathological symptoms and QoL in their early post-displacement context. This person-cantered approach combining psychopathology and QoL seems promising when studying trauma-affected refugees, as diagnosis-based methods often interpret refugees’ mental health through a psychopathological lens with a focus on individuals who meet predetermined diagnostic criteria. Whereas person-centered methods tend to account for the interplay of transdiagnostic symptoms in a holistic manner, potentially identifying individuals with both clinical and subclinical symptoms presentation, and those who diverge from commonly presented patterns of psychopathology and functioning. Moreover, the significant relationships between post-migration stressors such as insecure residence status and unemployment indicate how contextual factors may contribute to distinct patterns of suffering and functioning. A holistic approach to newly arrived refugees’ mental health should consider psychopathological symptoms, QoL, and post-migration factors in the assessment, service provision, and migration policies concerning this target group. While the study contributes to the existing body of knowledge by mapping out psychopathological symptoms and QoL in individuals with recent displacement experiences, there are several limitations.

First, the cross-sectional design of the study shows association rather than causality. Longitudinal research, investigating the developmental course of psychopathology and QoL in refugees upon arrival to resettlement will provide further insight into the temporal relations of pre-migration factors (e.g., PTEs) and post-migration distress to further distinguish individuals with natural recovery from those needing help. Second, the number of traumatic experiences of refugees and their influence on their profile membership were not explored due to lacking the variable. As a result, the impact of pre-migration traumatic events as possibly ongoing stressors versus post-migration difficulties on mental health and QoL of refugees remain unknown in this study. Third, the PTSD-dominant/Preserved QoL latent profile was described as resilient, this was rather arbitrary given the characteristics of this subgroup, this should not be interpreted as a directly observed response pattern as no resilience indictor was used in the analyses. Fourth, associations between latent profiles and socio-demographic variables were explored using most-likely class membership, without adjusting for classification error. Future research replicating our results using correction methods [97] is warranted. We used bivariate analyses for the associations between profiles and socio-demographic factors, therefore interpretations of our results should remain at exploratory level. We used a split-sample validation to assess the stability of our model, future research should add external validations into the process as well for more certainty regarding model stability and replicability. Finally, the data were collected through self-report outcome measures instead of diagnostic interview, although the measures were translated into participants’ native language following a rigorous iterative back and forth process, but self-report measures are susceptible to response biases. Therefore, the results in clinical settings should be interpreted with caution.

## Conclusions

Forcibly displaced populations are at high risk of mental health problems like PTSD, depression and anxiety due to exposure to trauma and other severe distress. Findings in the current study suggest that there are distinct subgroups based on symptoms severity and levels of QoL in individuals with recent refugee experiences. A unique subgroup of individuals identified in this study appears to be able to cope with mental health distress and maintain their resilience in spite of high PTSD symptoms. In the current study, individuals with Afghan nationality, without a residence permit, woman and unemployed were associated with severe levels of PTSD, depression and anxiety symptoms and low QoL. Discrepancies in mental health and QoL among refugee groups may be influenced by contextual factors such as residence status, rather than being solely a characteristic of the specific group itself. These results underscore the importance of appropriate assessments of subgroups with different patterns of psychopathological symptoms and QoL and adequate service planning for individuals who are in the process of resettlement in a new country, as one size may not fit all. The results also emphasize the detrimental impact of uncertain residence situation and unemployment on refugees’ mental health and advocate for adequate and effective migration policies.

## Supplementary Information

Supplementary Material 1.

## Data Availability

The de-identified data that support the findings of this study are available from the corresponding author upon reasonable request.

## Abbreviations

LPA: Latent Profile Analysis
LCA: Latent Class Analysis
PTSD: Post Traumatic Stress Disorder
QoL: Quality of Life
WHO: World Health Organization
PTEs: Potentially Traumatic Events
CMHD: Common Mental Health Disorders
