# Supplementary material for: Profiles of psychopathology and quality of life in individuals with recent displacement experiences: a latent profile analysis
Source: BMC Public Health. 2026 Apr 30;26:1424. doi: 10.1186/s12889-026-27604-w (PMC13130677; doi:10.1186/s12889-026-27604-w)
Supplement: Supplementary file 1 — Supplementary Material 1. [file 12889_2026_27604_MOESM1_ESM.docx]

**Categorization of Socio-Demographic Variables**

Eight socio-demographic indicators (age, gender, nationality education, employment status, housing situation, accompaniment and residence permit) were included in the analyses based on previous studies indicating their associations with psychopathology and QoL in refugee and host community populations. All variables except for age and nationality were dichotomized, and categories with few participants were collapsed into sufficiently large appropriate categories. Given that age was reported categorically, three categories (18-25 years, 26-35 years, and 36-65 years) were created, and 3 individuals (66 years and older) were labeled as missing. Gender was reported on a spectrum of “male”, “female” and “other”. To allow dichotomization, 7 individuals who reported their gender as “other” were marked as missing.

The education variable was dichotomized, and participants with a university level education and higher were labeled as “with higher education”, and other participants were collapsed into the category of “no higher education”. The employment status variable was dichotomized by collapsing “work” and “study” into one category and was labeled as “employed”, as well as participants who reported their employment status as “none” were marked as “unemployed”. The housing variable was dichotomized into “living in asylum accommodation*”* and “else”- indicating other types of accommodation such as owning a house, living in collective houses and other. Residence permit was reported as a dichotomized variable in the data set as “having a residence permit” and “not having a residence permit”. The accompaniment variable was dichotomized to “accompanied” accommodating all participants who reported that they had been accompanied by a friend, relative or family member when making the refugee journey and “unaccompanied”.

**Table S1**

*Intercorrelations Between WHOQOL-BREF Domains*

| Domains | 1 | 2 | 3 | 4 |
| --- | --- | --- | --- | --- |
| 1. Physical health | - | .68^***^ | .55^***^ | .61^***^ |
| 2. Psychological |  | - | .57^***^ | .55^***^ |
| 3. Social relationships |  |  | - | .56^***^ |
| 4. Environment |  |  |  | - |

Note*. WHOQOL-BREF* World Health Organization Quality of Life—brief version. ^***^*p* < .001

**Table S2**

|  | PHQ-9 | GAD-7 | PC-PTSD | WHOQOL-BREF |
| --- | --- | --- | --- | --- |
| Profile 1 vs. profile 2 | -3.64*** | -3.99*** | -2.36*** | 3.26** |
| Profile 1 vs. profile 3 | -9.96*** | -12.71*** | -2.60*** | 10.77*** |
| Profile 2 vs. profile 3 | -6.32*** | -8.71*** | -0.24* | 7.51*** |

*Pairwise Comparisons of the three Latent Profiles based on Psychopathological Symptoms Severity and Quality of Life - Mean Differences and Significance*

Abbreviations: *PHQ-9* Patient Health Questionnaire 9-item scale, *GAD-7* Generalized Anxiety Disorder 7-item scale, *PC-PTSD* Primary Care PTSD, *WHOQOL-BREF* World Health Organization Quality of Life—brief version. **p* < .05, ***p* < .01, ****p* < .001

**Table S3**

*Fit Indices for the Six Tested Models with Split-Samples (70% & 30%)*

| Number of  profiles | Sub-sample | AIC | BIC | SABIC | Entropy | Prob  min | Prob  max | N  min | N  max | BLRT_  p |
| --- | --- | --- | --- | --- | --- | --- | --- | --- | --- | --- |
| 1 profile | 70% | 3358.637 | 3412.295 | 3368.511 | 1.00 | 1.00 | 1.00 | 1.00 | 1.00 | NA |
|  | 30% | 1560.741 | 1603.167 | 1558.857 | 1.00 | 1.00 | 1.00 | 1.00 | 1.00 | NA |
| 2 profiles | 70% | 3226.109 | 3399.786 | 3339.509 | 0.71 | 0.89 | 0.92 | 0.41 | 0.58 | .009 |
|  | 30% | 1531.290 | 1588.868 | 1528.732 | 0.85 | 0.91 | 0.98 | 0.32 | 0.67 | .009 |
| 3 profiles | 70% | 3246.582 | 3339.648 | 3263.509 | 0.75 | 0.82 | 0.92 | 0.29 | 0.37 | .009 |
|  | 30% | 1507.600 | 1580.331 | 1504.369 | 0.88 | 0.88 | 0.99 | 0.30 | 0.37 | .009 |
| 4 profiles | 70% | 3245.574 | 3358.028 | 3266.027 | 0.68 | 0.57 | 0.91 | 0.14 | 0.29 | .188 |
|  | 30% | 1521.600 | 1609.483 | 1517.696 | 0.83 | 0.86 | 0.92 | 0.05 | 0.34 | .752 |
| 5 profiles | 70% | 3236.036 | 3367.89 | 3260.015 | 0.75 | 0.68 | 0.89 | 0.03 | 0.30 | .059 |
|  | 30% | 1531.609 | 1634.644 | 1527.032 | 0.76 | 0.75 | 0.90 | 0.09 | 0.26 | .683 |
| 6 profiles | 70% | 3227.436 | 3378.668 | 3254.942 | 0.75 | 0.66 | 0.89 | 0.03 | 0.30 | .039 |
|  | 30% | 1532.314 | 1650.501 | 1527.063 | 0.82 | 0.66 | 0.95 | 0.05 | 0.30 | .297 |

Abbreviations: *AIC* Akaike information criterion, *BIC* Bayesian information criterion, *SABIC* sample size-adjusted Bayesian information criterion, *Prob min/max*, the average latent class probability for most likely class membership by assigned class, *N min*/*max* proportion of the sample assigned to the smallest or largest classes, *BLRT_p* P-value for the bootstrapped likelihood ratio test

**Fig. S1**

*The Bayesian Information Criterion (BIC) Plot for Fourteen Different Covariance Parametrizations across Multiple Latent Profile Solutions and the Nine Mixture Components Used for Profile Selection.*


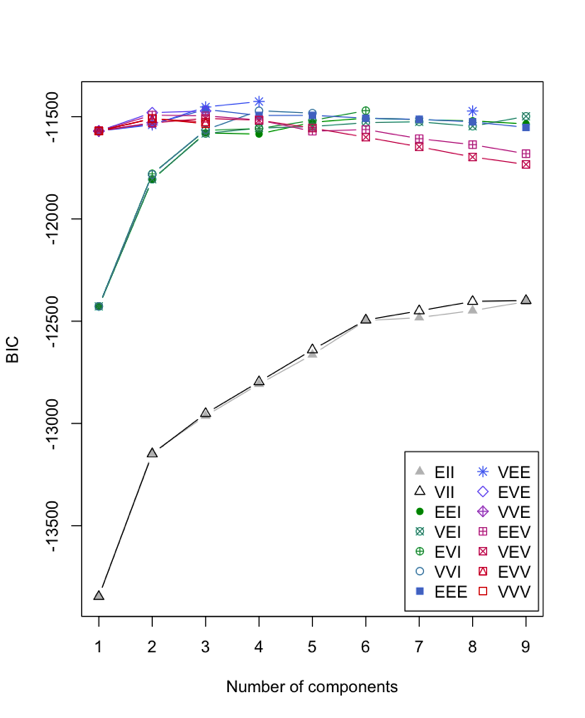


*Note*. Model abbreviations denote the geometric characteristics of the variance-covariance structure, including volume, shape, and orientation. EII (equal volume, identity shape, identity orientation), VII (variable volume, identity shape, identity orientation), EEI (equal volume, equal shape, identity orientation), VEI (variable volume, equal shape, identity orientation), EVI (equal volume, variable shape, identity orientation), VVI (variable volume, variable shape, identity orientation), EEE (equal volume, equal shape, equal orientation), VEE (variable volume, equal shape, equal orientation), EVE (equal volume, variable shape, equal orientation), VVE (variable volume, variable shape, equal orientation), EEV (equal volume, equal shape, variable orientation), VEV (variable volume, equal shape, variable orientation), EVV (equal volume, variable shape, variable orientation), and VVV (variable volume, variable shape, variable orientation). For further details, see Scrucca et al. (2023, pp. 13–18)

**Fig. S2**

*
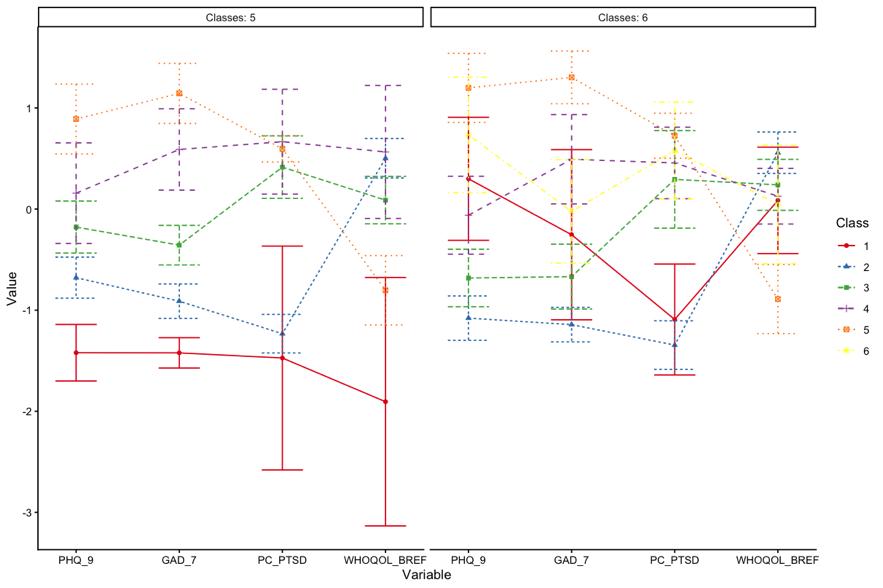
Profile Plots for Five- and Six-profile Models*

Abbreviations: *PHQ-9* Patient Health Questionnaire 9-item scale, *GAD-7* Generalized Anxiety Disorder 7-item scale, *PC-PTSD* Primary Care PTSD, *WHOQOL-BREF* World Health Organization Quality of Life—brief version

**Table S4**

| Variable | Mild Psy/High QoL | PTSD-dominant/Preserved QoL | Severe Psy/Low QoL |
| --- | --- | --- | --- |
| Age | | | |
| 18-25 | **-2.3** | 1.2 | 1.0 |
| 26-35 | 0.7 | 1.1 | -1.7 |
| 36-65 | 1.6 | **-2.5** | 0.8 |
| Gender | | | |
| Female | 0.2 | **-2.3** | **2.1** |
| Male | -0.2 | **2.3** | **-2.1** |
| Employment status | | | |
| Employed | 1.8 | 0.7 | **-2.4** |
| Unemployed | -1.8 | -0.7 | **2.4** |
| Residence permit | | | |
| No RP | **-6.3** | **2.4** | **3.7** |
| RP | **6.3** | **-2.4** | **-3.7** |
| Nationality | | | |
| Afghanistan | **-6.3** | 1.4 | **4.7** |
| Syria | **4.1** | 0.6 | **-4.5** |
| Iraq | -0.5 | 0.7 | -0.2 |
| Iran | -0.3 | -1.1 | 1.4 |
| Eritrea | **3.7** | -1.9 | -1.7 |
| Somalia | -0.2 | 1.5 | -1.3 |
| Other ^a^ | **2.2** | **-2.1** | 0.0 |

*Standardized Adjusted Residuals for Significant Socio-Demographic Variables based on Their Profile Membership*

Note. Mild Psy/High QoL Mild Psychopathology/High Quality of Life, Severe Psy/Low QoL Severe Psychopathology/ Low Quality of Life, RP residence permit, no RP no residence permit. Bolded positive residuals indicate that the observed frequency in the group is significantly higher than expected, whereas negative residuals indicate that the observed frequency is significantly lower than expected. ^a^ Other nationality groups (N < 5)

**Table S5**

*Percentages and Standardized Adjusted Residuals (SAR) for the Nationality Variable based on Residence Permit*

| Nationality | No residence permit | Residence permit |
| --- | --- | --- |
| Afghanistan | 189 (96.4%) | 7 (3.6%) |
| SAR | **9.7** | -9.7 |
| Syria | 56 (40.9%) | 81 (59.1%) |
| SAR | **-9.5** | 9.5 |
| Iraq | 49 (96.1%) | 2 (3.9%) |
| SAR | **4** | -4 |
| Iran | 21 (95.5%) | 1 (4.5%) |
| SAR | **2.5** | -2.5 |
| Eritrea | 6 (28.6%) | 15 (71.4) |
| SAR | **-4.5** | 4.5 |
| Somalia | 10 (90.9%) | 1 (9.1%) |
| SAR | 1.4 | -1.4 |
| Other^a^ | 36 (50%) | 36 (50%) |
| SAR | **-4.5** | 4.5 |

*Note*. Bold-faced positive residuals indicate that the observed frequency in the group is significantly higher than expected, whereas negative residuals indicate that the observed frequency is significantly lower than expected. ^a^ Other nationality groups (*N* < 5)
